# Unveiling Solvent Effects on β-Scissions through Metadynamics and Mean Force Integration

**DOI:** 10.1021/acs.jctc.4c00383

**Published:** 2024-07-03

**Authors:** Francesco Serse, Antoniu Bjola, Matteo Salvalaglio, Matteo Pelucchi

**Affiliations:** † Department of Chemistry Materials and Chemical Engineering, Politecnico di Milano, Piazza Leonardo da Vinci 32, Milan 20133, Italy; ‡ Thomas Young Centre and Department of Chemical Engineering, University College London, London WC1E 7JE, U.K.

## Abstract

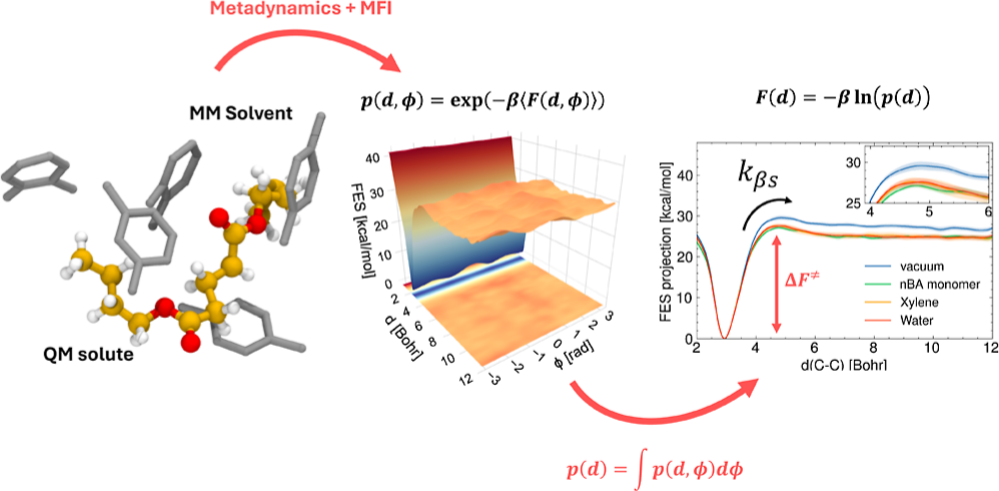

This study introduces a methodology that combines accelerated molecular dynamics and mean force integration to investigate solvent effects on chemical reaction kinetics. The newly developed methodology is applied to the β-scission of butyl acrylate (BA) dimer in polar (water) and nonpolar (xylene and BA monomer) solvents. The results show that solvation in both polar and nonpolar environments reduces the free energy barrier of activation by ∼4 kcal/mol and decreases the pre-exponential factor 2-fold. Employing a hybrid quantum mechanics/molecular mechanics approach with explicit solvent modeling, we compute kinetic rate constants that better match experimental measurements compared to previous gas-phase calculations. This methodology presents promising potential for accurately predicting kinetic rate constants in liquid-phase polymerization and depolymerization processes.

## 1 Introduction

Free radical polymerization (FRP) and pyrolysis processes are key technologies with wide-ranging applications in fields such as material synthesis and chemical recycling. Understanding the underlying reaction mechanisms and assessing the impact of the chemical environment on reaction kinetics is crucial for the design and scale-up of such processes. In particular, the ability to predict kinetic parameters of elementary reactions in solution or in polymer bulk is key for formulating reliable mechanistic hypotheses describing the dynamics of complex reactive environments. To date, kinetic rate parameters of FRP can be accessed experimentally via pulsed laser polymerization (PLP) coupled with size exclusion chromatography (SEC)^1,2^ as well as by performing semibatch solution polymerization coupled with nuclear magnetic resonance (NMR).^3^ The experimental determination of rate constants is constrained by the operating capabilities of laboratory-scale reactors and diagnostics, thus inhibiting the exploration of wider ranges of temperatures (*T* > 400 K) and limiting the combinations of solvents and mixture composition typical, within others, of thermal depolymerization processes. The main reaction classes involved in polymerization processes are known to be propagations and terminations of end chain radicals (ECR) by recombination or disproportionation. ECRs are formed starting from the reaction of a radical initiator (usually a peroxide) with a monomer. In addition, at increasing temperatures, a large proportion of polymer chains (poly ethylene, polystyrene, poly butyl acrylate [PBA], etc.) exhibit a high propensity to form midchain radicals (MCRs) through intramolecular (or less likely intermolecular) hydrogen abstraction, also known as backbiting. These MCRs can then undergo β-scission, leading to a reduction in the average polymer chain length. When monomer concentration is sufficiently high, MCRs can add to monomers, leading to branched chains. The formation of MCRs becomes more relevant at temperatures >410 K^3,4^ and, therefore, is mainly present in pyrolysis, which is carried out well above this temperature (i.e., >600 K), and thermally self-initiated polymerization processes.

Most experimental studies reporting elementary kinetic rate constants for radical reactions in the liquid phase are focused on polymerization technology, thanks to the milder operating conditions and higher selectivity compared to pyrolytic depolymerization conditions. Moreover, these experimental studies are driven by the need to find relationships between operating conditions and final polymer quality and structure. In contrast, in the context of pyrolysis, kinetic parameters are mostly estimated using empirical relationships.^5−7^ These aspects significantly limit the availability of experimental data of elementary rate parameters at high temperatures, which substantially limits the understanding and, in turn, the design of technological applications of self-initiated polymerization as well as depolymerization processes.

The experimental elementary rate parameters that can be taken as references for the validation of computational predictions are those derived according to the standards of the IUPAC PLP–SEC database.^8^ PLP studies mainly focus on determining propagation coefficients in FRP conditions. The propagation coefficients derived from PLP have then been used for deriving backbiting and MCR propagation (also known as short-chain branching) kinetic rate coefficients.^4,9^ A recent study proposed the application of PLP–SEC at temperatures higher than the ones typical of polymerization conditions (>400 K) for determining the β-scission of MCRs.^4,10^ However, despite a general consensus on the validity of PLP–SEC measurements at low temperatures (<400 K), these high-temperature (>400 K) applications^4,10^ are yet to achieve widespread adoption. A more typical approach for estimating kinetic parameters that can also be used for validation is by regressing experimentally obtained average molecular weight and NMR data to detailed kinetic models describing the reactivity of both ECRs and MCRs in a conventional semibatch setup. However, a significant discrepancy can be observed between the high-temperature PLP–SEC and the more conventional techniques.

In this paper, we develop and test a generally applicable computational protocol for the determination of elementary rate constants in an explicitly modeled condensed phase with the aim of improving our understanding of the physical chemistry of liquid phase chemical reactivity and, at the same time, reducing the need for expensive experimental campaigns. The development of such a theoretical methodology requires careful and extensive validation against kinetically reliable experimental data. In this regard, acrylate polymers have been the subject of many experimental studies spanning from conventional semibatch solution polymerization to PLP–SEC at high and low temperatures, thanks to their widespread technological applications as coatings. For this reason, butyl acrylate (BA) polymerization has been selected as an ideal validation target for our newly developed theoretical methodology. In particular, this work focuses on the β-scission of PBA’s MCRs. The computational approach followed in this work is based on a hybrid quantum mechanics/molecular mechanics scheme (QM/MM), as implemented in CP2K 9.1.^27^ This approach significantly reduces the computational cost of the simulation by adopting a classical force field for treating the solvent while preserving the expensive quantum mechanical electronic structure calculations only for the subset of the system undergoing a chemical transformation. Recent studies are starting to overcome the QM/MM approach in favor of ad hoc machine-learned potentials based on density functional theory (DFT). Nevertheless, QM/MM approaches can still deliver key insights without the need to generate a large dataset and train a neural network for each specific system.

We apply QM/MM to estimate the free energy landscape associated with β-scission reactive events using metadynamics.^11,12^

Metadynamics is an enhanced sampling technique based on introducing an adaptive bias potential to a selected subset of degrees of freedom of a system, allowing the sampling of physical-chemical phenomena associated with long time scales. In this context, the adjective long refers to time scales that exceed the characteristic times practically accessible by a brute-force propagation of the system dynamics.

The reactive trajectories are sampled with standard metadynamics and reweighted using mean force integration (MFI).^13^ Using metadynamics combined with MFI, we obtain a converged estimate of the free energy surface from multiple independent short simulations that can run in parallel, thus significantly increasing computational efficiency. Despite the significant computational cost savings introduced by this methodology, performing accurate electronic structure calculations, even at the DFT level, is still too expensive to be applied to dynamic calculations. Therefore, a compromise must be found between accuracy and computational cost.

**Figure 1:**
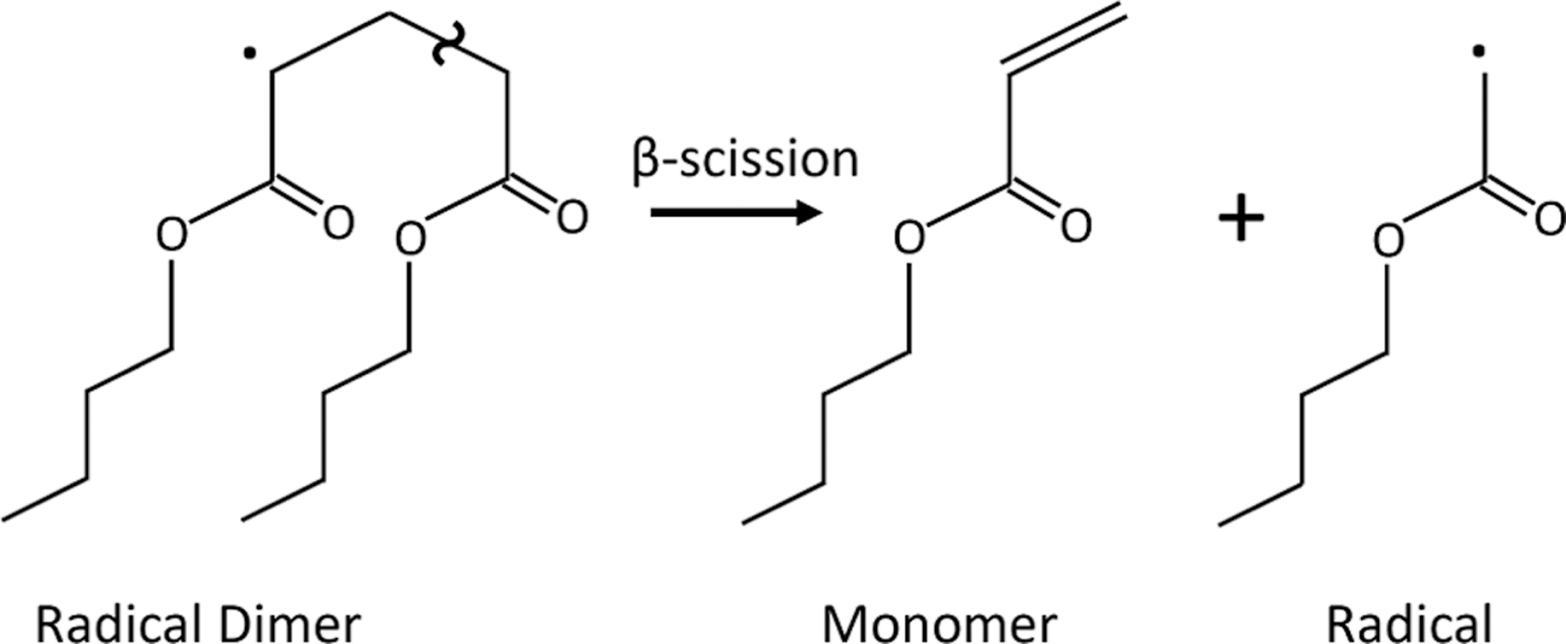
Model reaction for PBA’s β-scission.

The simulation of β-scission is carried out on model radical dimers (Figure 1) in vacuum, in xylene solvent, bulk (BA monomer), and in water. The calculations performed in vacuum serve, on the one hand, as a benchmark with respect to previous computational results in gas-phase from the literature^14^ and, on the other hand, to unveil possible solvent effects on the reactivity. Here, we assume that small model oligomers can well represent the reactivity of the polymer chains. This assumption is based on the hypothesis that reactivity is affected by the local electronic structure only and not by the length of the polymer backbone.^15−18^ To obtain quantitative estimates of the rate constants for the β-scission, we have employed the rare events generalized transition state theory (TST), which has been proven to be a reliable method for calculating reaction rates in complex systems^19^ without the need to calculate the partition functions explicitly. In fact, state-of-the-art computational techniques based on DFT calculations and harmonic TST (HTST), which neglect the interactions with the solvent, significantly underestimate the experimental kinetic rate measurements.^14^ The insights gained from our computational approach can guide experimental efforts and provide a deeper understanding of the role of solvents on the reaction kinetics and thermodynamics of radical chain mechanisms.

## 2 Theory and Methods

### 2.1 Rare Event Sampling: Metadynamics

Metadynamics is a molecular simulation technique that accelerates the sampling of rare events by biasing the system with a history-dependent potential that enhances the fluctuation of a selected set of degrees of freedom (i.e., collective variables-CVs). This technique allows sampling phenomena whose time scales are hardly accessible with conventional molecular dynamics, such as chemical reactions, typically in the order of μ-seconds (10^–6^). The CVs considered in this study are simple functions of the coordinates of the system (distances, angles, and dihedral angles) sufficient for describing the chemical–physical phenomenon investigated. In this work, we used standard metadynamics^20,21^ to study the solvent effects on the β-scission of PBA. Standard metadynamics is the simplest variant of its kind, and it has two main advantages over the more popular well-tempered metadynamic (WTMTD) variant.^11,12^ The first advantage is that standard metadynamics allows a faster exploration of the free energy landscape because the amplitude of the spawned bias functions does not reduce as the system progresses toward the metastable states. Furthermore, the second advantage is that this technique does not require a-priori knowledge of the amplitude of the free energy barrier being investigated, but it can be used to explore unknown free energy landscapes. The major downside of standard metadynamics is that it does not converge to a specific solution within a single simulation.^11^ However, this issue can be overcome by setting a threshold once the phenomenon being investigated has been sampled (e.g., the free energy barrier of the activation of chemical reactions). Nevertheless, a faster exploration of the phase space also implies that the system spends a smaller amount of time in each sampled state, yielding a higher sampling error with respect to the WTMTD variant. The sampling error can be systematically reduced by averaging multiple independent simulations. This averaging procedure is allowed by the MFI algorithm,^13^ which eliminates the problem of aligning the free energies from independent metadynamics simulations caused by the presence of the ensemble average of the total bias accumulated in each simulation.^13^

### 2.2 Simulation Details

All the simulations have been performed using the CP2K 9.1 software. We used the generalized Amber force field (GAFF) for the initial equilibration of the system in a periodic box and to model solvent molecules. The molecular dynamics time step is 0.5 fs in all simulations. The Nose–Hoover^22,23^ thermostat with a time constant of 50 fs is used to maintain constant temperature over the equilibration runs, whereas for the metadynamics runs as well as the unbiased trajectories for performing the histogram test, we employed the Bussi–Parrinello thermostat.^24^ Furthermore, long-range electrostatic interactions are accounted for with the smooth particle mesh Ewald method with a real space cutoff of 10 Å. Overall, the equilibration step has been subdivided into a preliminary 100 ps run at constant pressure and temperature (*NPT*), which is required for achieving the equilibrium liquid bulk density. Once the volume of the periodic box has reached convergence, an additional 50 ps equilibration run at constant volume (*NVT*) is performed.

**Figure 2:**
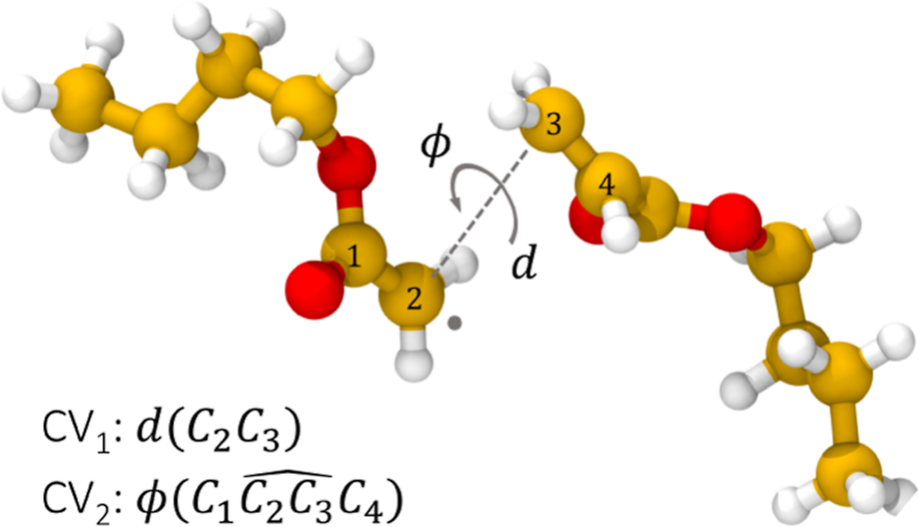
Definition of the CV space for the β-scission.

For the metadynamic simulations of the β-scission reactions, a QM/MM scheme has been adopted. The QM region is composed of the dimer undergoing β-scission. The potential energy of the QM region is switched from GAFF to GFN1 extended tight binding Hamiltonian (GFN1-xTB^25^) because of its low computational cost, reasonable accuracy for calculating equilibrium structures, vibrational frequencies, and noncovalent interactions of large systems. However, the accuracy of such a tight binding method toward energy barriers of activation has not yet been established.^25^ The coupling between the QM and MM regions is accounted for using a Coulomb potential.^26,27^ Before the actual metadynamics run, a preliminary *NVT* equilibration of 1 ps is performed to thermalize the system with the new potential energy partition. The CVs subjected to the bias are the distance between the atoms involved in the breaking bond (*d*_C–C_) during the reaction and the dihedral angle (ϕ) between the two moieties of the dimer that undergoes β-scission, as shown in Figure 2. The bias potential is updated with Gaussians having a height of 1 kcal/mol and a width of 0.2 Bohr, deposited every 60 timesteps as a function of both CVs.

### 2.3 Free Energy Surface from MFI

The free energy landscape is recovered from the metadynamics simulations through the MFI algorithm implemented in the pyMFI package.^13,27^ This implementation of the MFI reconstructs an analytical expression for the mean thermodynamic force in the CV space ∇*F*_*t*_(**s**) up to a given time *t*, where *F*_*t*_(**s**) refers to the Helmholtz free energy. As reported in ref (13), the mean force has two components, namely, the gradient with respect to **s** of the natural logarithm of the biased probability density *p*_*t*_^b^(**s**) and the gradient of the bias potential *V*_*t*_(**s**) accumulated up to time *t*, as shown in eq 1
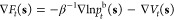
1

The biased probability density *p*_*t*_^b^(**s**) is reconstructed in the time interval between each update of the bias potential *V*_*t*_(**s**) as the sum of bivariate Gaussian kernels centered in **s**(*t*) = (d(*t*), ϕ(*t*)) (eq 2), which is the vector of the selected CVs at time *t*, allowing also to derive an analytical expression for its derivative with respect to **s**(13)
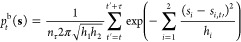
2where *n*_τ_ is the number of values of each CV stored between each bias potential update. In our case, the CV values are saved every 10 steps, and a Gaussian bias is spawned every 60 steps; therefore, *n*_τ_ equals 6. The bandwidths of the Gaussian kernels *h*_*i*_ are set to 0.2, the same width of the Gaussians constructing the metadynamic bias potentials. In addition, the analytical expression of the bias potential accumulated up to time *t* in a standard metadynamics simulation, *V*_*t*_(**s**), can be expressed as in eq 3
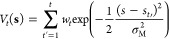
3

Where the height of each bias function *w*_*t*_ is constant throughout the simulation for standard metadynamics and has been set equal to 1 kcal/mol, whereas σ_M_, the bandwidth of the bias, is equal to 0.2, as mentioned in Section 2.1. Since the mean force is independent of the history of the accumulated bias in each simulation (i.e., the ensemble average of the bias potential),^13^ this methodology allows the merging of the mean force estimates from independent simulations into a single refined estimate of the free energy. As a result, the convergence can be significantly accelerated because the mean force estimate can be obtained by patching multiple independent simulations of short duration instead of performing a single long simulation. The sampling error of the free energy estimate is performed by bootstrapping across the independent samples. The bootstrap variance of the free energy in the CV space (eq 4) is computed with respect to the weighted average over the biased probability density (eq 5). Therefore, the Bessel correction must be applied 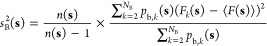
4
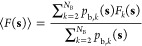
5
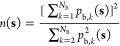
6

The quantity *n*(**s**) defined in eq 6 is the effective sample size used to compute the Bessel correction. It is worth noticing that the free energy surface must be computed by integration of the mean thermodynamic force at each *k*-th iteration of the bootstrap procedure. For this reason, the integration has been performed by means of the fast Fourier transform,^27^ allowing for a substantial gain in computational efficiency. In this work, the number of bootstrap iterations (*N*_B_) was set to 500, which is sufficient to converge the bootstrap variance.

The free energy barrier of activation has been calculated from *F*(*d*_C–C_), the free energy profile along the C–C distance (i.e., CV_1_ in Figure 2) obtained by integrating out the normalized probability density *p*(*d*_C–C_, ϕ), along ϕ (i.e., CV_2_ in Figure 2) as
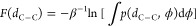
7where
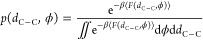
8

In eq 8, ⟨*F*(*d*_C–C_, ϕ)⟩ indicates the bootstrap average of *F*(*d*_C–C_, ϕ) obtained via eq 5. The error associated with *F*(*d*_C–C_) is estimated following a bootstrapping procedure according to eq 4.

This protocol allows, on the one hand, to simplify the calculation of the free energy barrier of activation of the chemical reaction by projecting the free energy on just one effective dimension and, on the other hand, to ensure that the orthogonal degrees of freedom (i.e., the torsional angle ϕ) are completely sampled via the metadynamic multivariate bias. Once the free energy profiles are determined at different temperatures, it is possible to decouple the enthalpic and entropic contributions as a function of the distance by linear regression. This decoupling allows for correcting the xTB electronic energy with higher-level electron correlation methods. In this work, the enthalpy profile Δ*H*(*d*) has been corrected by optimizing the geometries and vibrational frequencies of snapshots taken from the metadynamic trajectory using the DFT functional ωB97XD/def2-TZVPP^28^ in vacuum, yielding the difference in internal energy between reactants and transition states, including the zero-point vibrational energy (ZPE) correction (eq 9). Then, the free energy barriers of activation used for the calculation of the rate parameters in the generalized TST framework have been obtained as in eq 10
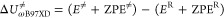
9
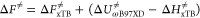
10

This protocol enables an optimal compromise between computational cost, using the cheaper xTB for metadynamic configurational sampling, and accuracy, using state-of-the-art DFT molecular energies (within 2–3 kcal/mol accuracy^29^) only for a few frames along the reactive trajectory.

### 2.4 Histogram Test

While in static calculations the transition state is distinctively determined as a saddle point on the potential energy surface, in dynamic calculations this is not the case as the free energy is projected on the CV space, which is a low dimensional representation of the actual phase space. Therefore, it is necessary to test if this low dimensional representation **s** is sufficient for capturing the dividing surface (also referred to as a dynamic bottleneck) or if it is necessary to increase its dimensions. This test can be performed by launching unbiased trajectories initialized in the metastable states that have been found on the free energy surface, which means, in our case, initializing simulations at the distance of the breaking bond that maximizes the minimum free energy pathway and computing the distribution of the probability of falling toward reactant or product states. This probability is also known as the committor probability. When the resulting distribution is unimodal and has a sharp peak for a committor probability equal to 0.5, the chosen CVs are sufficient for representing and sampling the dynamic bottleneck. Otherwise, other descriptors should be included to capture the dividing surface correctly.^30,31^

### 2.5 Evaluation of Reaction Rates

The free energy surface is not sufficient for obtaining estimates of kinetic rates, but it only gives information about thermodynamic equilibrium between given states of the system. In this work, the expression for the rate constants is derived from the rare events generalized TST. First, the phase space is subdivided into two regions, which can be identified as reactant (R) and product (P) states. Then, the rate constant  can be expressed as the average net flux from reactants to products (for a detailed derivation of 11 the reader is referred to refs^19 and 32^)
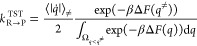
11

The integral at the denominator has the dimensions of a distance (Å) and stands for the length of the reaction coordinate, here assumed to be one-dimensional, connecting the reactant state to the transition state. This term has been derived from the integral over the set Ω of all possible values of the reaction coordinate (*q*), which in this case coincides with the C–C distance (*d*) times a step function *h*_R_, which assumes the value 1 if *q* < *q*_≠_ and 0 otherwise, as shown in eq 12
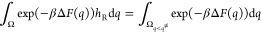
12

## 3 Results and Discussion

### 3.1 Free Energy Surface Exploration

**Figure 3:**
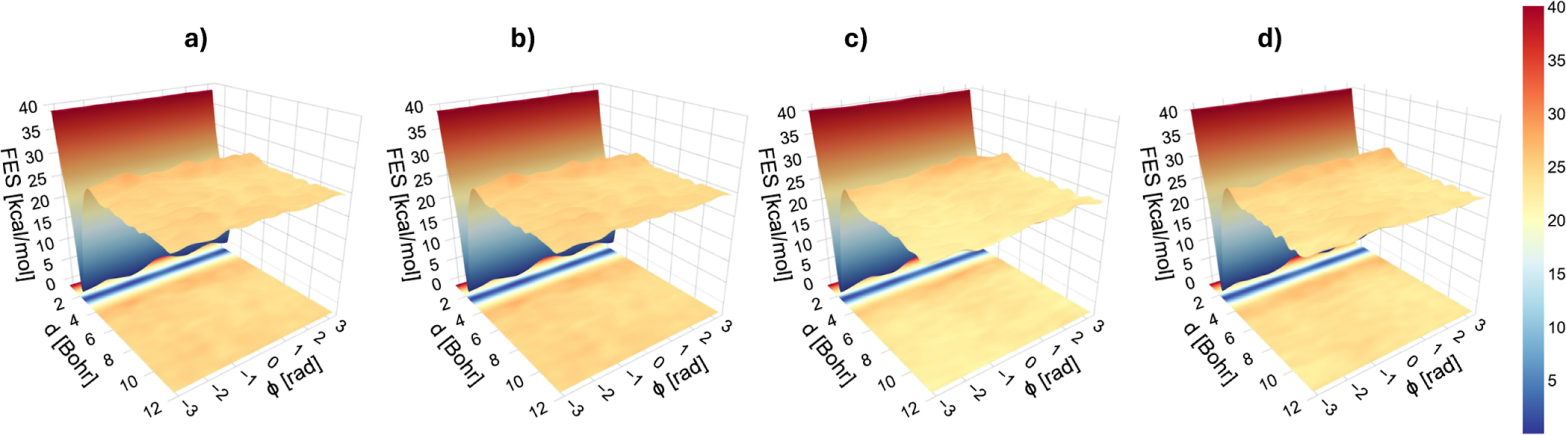
(a–d) Free energy surfaces resulting from the MFI of the bivariate (distance *d*_C–C_ and dihedral angle ϕ) metadynamics sampling at *T* = 410 K in (a) vacuum, (b) xylene, (c) water, and (d) BA monomer.

Figure 3a–d shows the Helmholtz free energy surfaces defined on the subspace of CVs consisting in the distance of the breaking bond (*d*_C–C_) and the angle (ϕ) between the planes intersecting in *d* (Figure 2) for the β-scission of the BA dimer in vacuum and under the effect of nonpolar (i.e., BA monomer and xylene) and polar (i.e., water) solvents. The height of the free energy barrier of activation of the chemical reaction, along the C–C distance, is shown to be approximately seven times higher than the barriers for torsional transitions along ϕ in the reactant state (i.e., the bounded dimer). This suggests that torsional transitions in the bounded state proceed orders of magnitude faster than the β-scission and, therefore, achieve thermodynamic equilibrium before the reaction takes place, allowing for a complete decoupling between the two dimensions (*d*, ϕ). In all the conditions investigated, the torsional free energy profile in the bounded state displays three equivalent minima, suggesting that the solvent does not significantly affect these equilibrium conformations. This result is corroborated by the study of the bivariate bootstrap variance, as reported in Figure S1 of the SM. Two distinct channels show a faster convergence of the bootstrap variance, which, being weighted by the biased probability density *p*_b_(**s**) (eq 4), is small (within 1–2 kcal/mol^2^) for the configurations that are more frequently explored. In contrast, unexplored regions show a much larger variance (>3 kcal/mol^2^).

**Figure 4:**
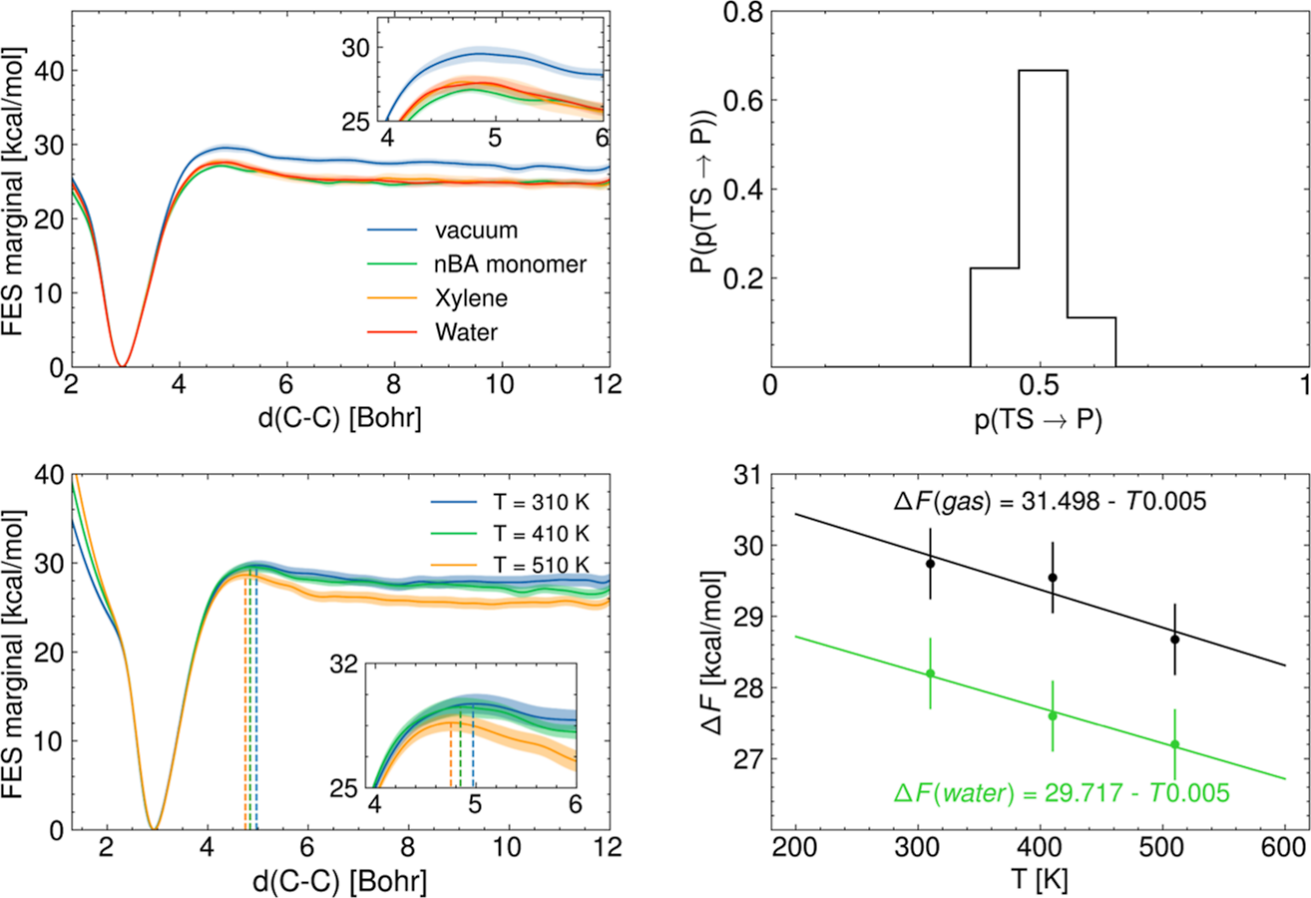
(a) Marginal free energy profiles along the C–C distance in the gas phase and in solution (xylene, BA monomer, and water); (b) distribution of the committor probability for performing the histogram test for the β-scission in the gas phase at *T* = 410 K; (c) marginal free energy profiles in the gas phase at *T* = 310, 410, and 510 K; and (d) free energy barriers of activation in vacuum (black) and in water (green) as a function of temperature together with a linear fit showing the entropy (slope) and enthalpy (intercept) of activation of the β-scission.

**Figure 5:**
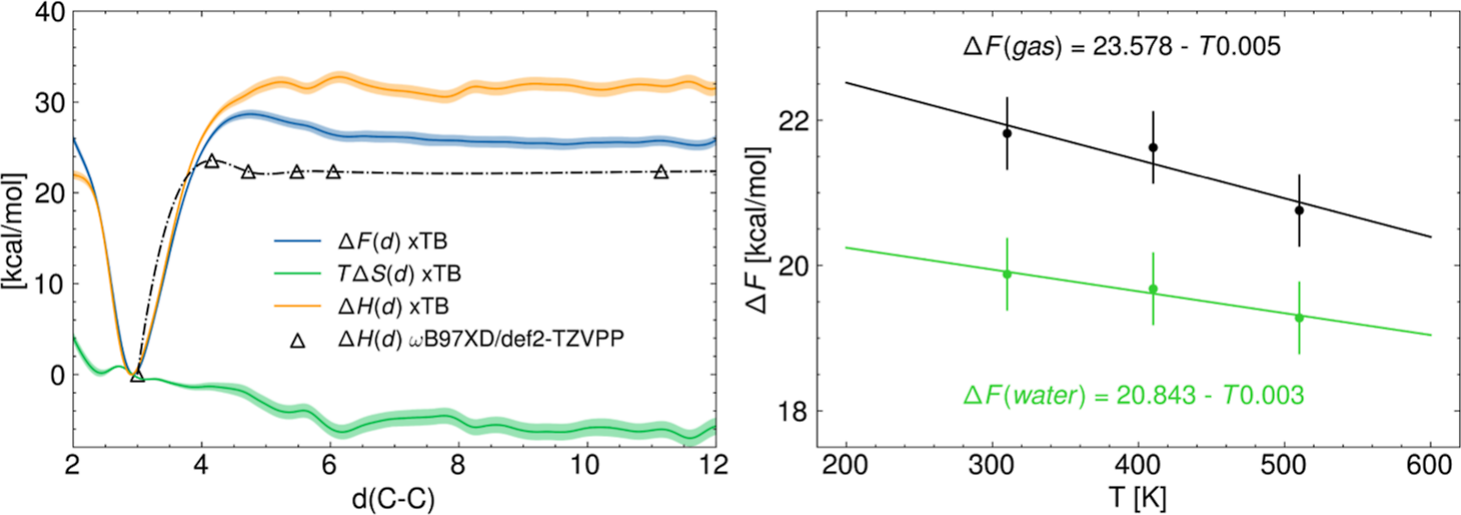
Left: decomposition of the free energy profile (gas phase) into its enthalpic and entropic components. Blue line is the free energy profile along *d*_C–C_ at *T* = 510 K, the green line is the entropy profile, and the yellow line is the enthalpy profile. The black dash-dotted line shows the internal energy profile plus the zero-point energy obtained by performing constrained optimizations as a function of the C–C distance using the DFT method ωB97XD/def2-TZVPP. Right: corrected free energy barriers of activation accounting for the zero-point energy from ωB97XD/def2-TZVPP at *T* = 310, 410, and 510 K in gas phase (black) and in water (green).

The marginal Helmholtz free energy profiles resulting from the marginalization over the angle ϕ are solely dependent on the distance *d*_C–C_, which has been selected as the effective reaction coordinate for the β-scission (Figure 4a). These profiles allow assessment of the impact of the different solvents on the free energy barriers of activation, which are used to calculate the kinetic rate constants in the generalized TST framework. It stands out from Figure 4a that the noncovalent interactions with the surrounding liquid environment induce a reduction of the free energy barrier of ∼4 kcal/mol. The bootstrap standard error of the marginal free energy allows the estimation of the uncertainty of the average free energy profile across the independent samples, which is between 0.45 and 0.60 kcal/mol. This implies that a significant discrepancy exists between the free energy barriers of activation in a vacuum and in the presence of the different solvents. While no significant discrepancy is observed between water and xylene solvents, the best estimate of the free energy barrier of activation obtained in simulations carried out in BA monomer, which is used in this study to represent the polymer bulk environment, is 0.5 kcal/mol smaller with respect to the case in water and xylene. Results from simulations at different temperatures are reported in Figure 4c,d, showing that the free energy barrier of activation and the free energy of reaction decrease at increasing temperatures, allowing us to estimate the enthalpy and entropy of activation^33,34^ (Figures 4d and 5) for the β-scission. In the gas phase, the enthalpy of activation obtained from the GFN1 xTB is Δ*H*_xTB_ = 31.5 ± 0.5 kcal/mol and the entropy of activation is Δ*S*_xTB_ = 0.0053 ± 0.0013 kcal/mol/K. Instead, in an aqueous environment, these contributions are Δ*H*_xTB_ = 28.7 ± 0.5 kcal/mol and Δ*S*_xTB_ = 0.0045 ± 0.0013 kcal/mol/K. In addition, the profiles show a slight reduction in the C–C distance associated with the dividing surface at increasing temperatures, meaning that at higher temperatures, the reactive flux increases due to both a decrease in the free energy barrier of activation and a narrowing of the dividing surface.

### 3.2 Histogram Test

The histogram test was performed by initializing unbiased trajectories at different torsional angles ϕ and at a C–C distance, corresponding to the free energy barrier of activation, around 4.7 Bohr. The results show a sharp peak of the committor probability distribution around a committor probability of 0.5 for the vacuum case (Figure 4b). This means, on the one end, that the metastable state found as a function of the distance *d*_C–C_ only is sufficient for the identification of the dividing surface and, on the other end, justifies identifying the potential of mean force, here calculated through the MFI, as the Helmholtz free energy.^35^

### 3.3 Potential Energy and ZPE Correction

Figure 5 shows the decoupling of the free energy profile into its enthalpy and entropy profiles for the case in vacuum. These profiles are obtained by linear regression of the free energy profile for each distance *d*_C–C_ at three temperatures *T* = 310, 410, and 510 K. The uncertainty of these profiles has been estimated with the position-dependent standard error for the coefficients of a two-parameter least-squares regression model, as shown in eqs 13 and 14
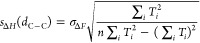
13
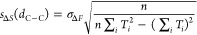
14where σ_Δ*F*_ is the square root of the sum of the free energy profiles bootstrap standard errors at the three temperatures considered and *n* is the number of temperature evaluations, thus *n* = 3. The enthalpy profile increases up to the C–C distance corresponding to the dividing surface, from which it reaches a plateau at approximately 31.5 kcal/mol. Entropy, on the other hand, decreases steadily; this means that the number of degenerate configurations increases as the two dimer fragments are separated up to a plateau. This indicates that after a certain distance, the number of degenerate configurations increases with the same proportionality as the phase space volume explored, thus yielding a null entropy change. The knowledge of the enthalpic contributions to the free energy profile allows us to perform a correction with the internal energy profile evaluated at *T* = 0 K with ωB97XD/def2-TZVPP for five snapshots extracted from the reactive trajectory as explained in the methods, Section 2.3. The correction factor evaluated from the trajectory in vacuum equals 7.92 kcal/mol and has also been applied to the cases in solvent. The corrected free energy barriers of activation Δ*F*_*d*_^≠^ as well as reaction Δ*F*_*d*_^R^ are reported in Table 1.

**Table 1:** Calculated Free Energy Barriers of Activation Δ*F*_*d*_^≠^ and Reaction Δ*F*_*d*_^R^, Frequency Factors Given by the Average Speed of Crossing the Dividing Surface Normalized by the Length of the Path Connecting the Transition State and the Free Energy Minimum *l*_*d*_ and Corresponding Rate Constants in the Gas Phase and Various Solvents (BA Monomer, Xylene, and Water)

solvent	temperature (K)	Δ*F*_*d*_^≠^ (kcal/mol)	(Bohr/s)	*l*_*d*_ (Bohr)	(s^–1^)	Δ*F*_*d*_^R^ (kcal/mol)
vacuum	310	21.8 ± 0.5	7.2 × 10^12^	2.0	0.0016 ± 0.0013	20.1 ± 0.5
vacuum	410	21.6 ± 0.5	7.2 × 10^12^	1.9	12 ± 7	19.1 ± 0.5
vacuum	510	20.8 ± 0.5	7.2 × 10^12^	1.8	5000 ± 2000	17.9 ± 0.5
BA monomer	410	19.1 ± 0.5	7.2 × 10^12^	1.9	300 ± 200	16.6 ± 0.5
xylene	410	19.6 ± 0.5	7.2 × 10^12^	1.9	200 ± 120	18.1 ± 0.5
water	310	20.1 ± 0.5	7.2 × 10^12^	1.8	0.03 ± 0.02	15.9 ± 0.5
water	410	19.6 ± 0.5	7.2 × 10^12^	1.7	150 ± 90	16.6 ± 0.5
water	510	19.5 ± 0.5	7.2 × 10^12^	1.6	20,000 ± 10,000	16.1 ± 0.5

### 3.4 Rate Constant Determination

The trajectories that have been used for performing the histogram test allow us to estimate the average speed of crossing the dividing surface , which in vacuum as well as in water assumes an average value of 7.2 × 10^12^ Bohr/s. Table 1 reports the average velocity of crossing the dividing surface in the presence and absence of solvent, calculated at *T* = 410 K only. The influence of temperature on the frequency factor is introduced by the changing distance *l*_*q*_ corresponding to the difference between the equilibrium C–C distance in the bonded state (reactant state) and the distance corresponding to the metastable state on the marginal free energy profile (see Table 1). As previously mentioned, this distance *l*_*q*_ decreases at increasing temperatures and is systematically lower by 0.2 Bohr/s in the cases with the various solvents, reflecting higher frequency factors. The average speed of crossing normalized by the distance *l*_*q*_ is approximately half the frequency factor that can be obtained from the HTST approximation *k*_B_*T*/ℏ, which at *T* = 410 K is equal to 8.5 × 10^12^ s^–1^. This result is consistent with the fact that the frequency factor in the HTST is an upper limit for the crossing frequency and becomes less accurate for phenomena characterized by high activation barriers, such as the β-scission here studied.^19^

**Figure 6:**
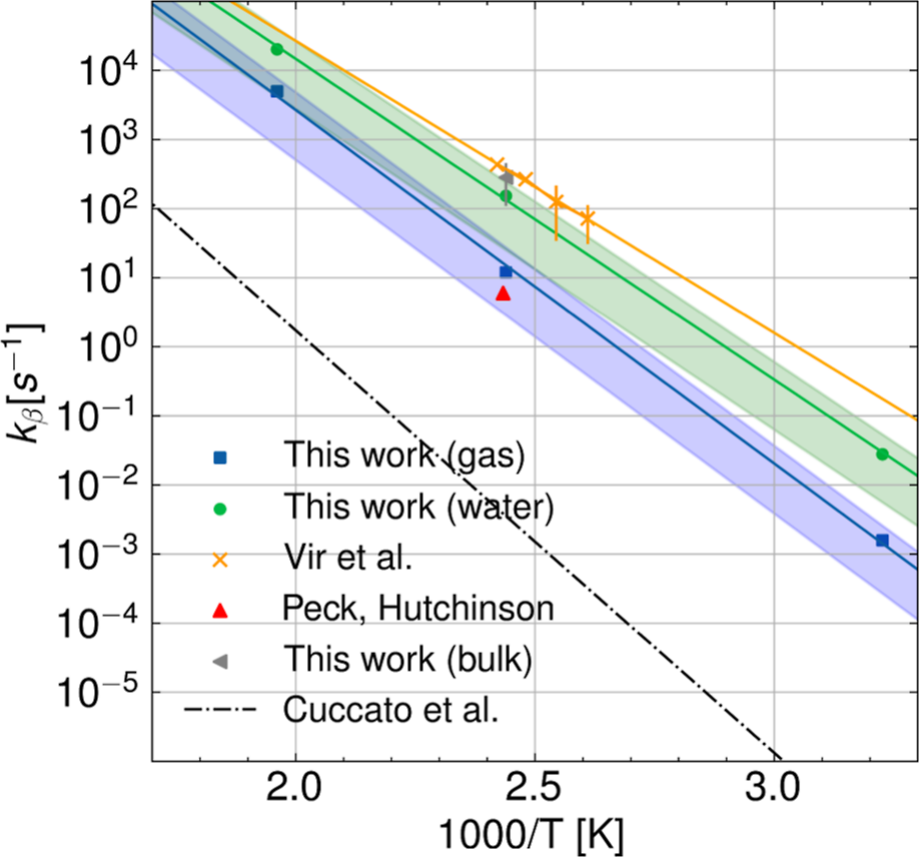
Arrhenius plot showing the comparison between calculated and experimental (from PLP–SEC and semibatch) kinetic rate constants for the different solvents. The experimental rate parameters from Vir et al.^4,10^ have been obtained in bulk, which in this study is modeled using the BA monomer as a solvent. Instead, the data from Peck et al.^3^ was obtained through semibatch experiments using xylene as a solvent.

The zero-point energy-corrected free energy barriers of activation and frequency factors are used to compute rate parameters through the generalized TST. For the cases in vacuum and water, the knowledge of rate parameters at three different temperatures *T* = 310, 410, and 510 K allows us to derive Arrhenius relationships, which are reported in eqs 15 and 16
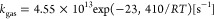
15
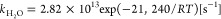
16where the gas constant *R* is in cal/mol/K. In addition, the simulations carried out in bulk (BA monomer) and xylene performed only at *T* = 410 K allow us to assess the effect of different solvents on the kinetic parameters. In particular, xylene solvent exerts the same effect as water solvent at 410 K. In contrast, BA monomer causes a 2-fold increase in the kinetic parameter and agrees within a factor of 1.5 with the experimental determination of Vir et al.^4^Figure 6 shows the Arrhenius correlations obtained from the rare events TST in the gas phase and water compared to computational predictions from the literature^14^ and experimental values.^3,4,10^ The best estimates for the activation energies obtained in the gas phase (blue squares and blue lines) and in the water solvent (green circles and green lines) were 23.41 and 21.24 kcal/mol, respectively. The result in bulk (gray triangle) at 410 K is also reported, whereas the result in xylene is not shown because it overlaps with the result in water at 410 K. Our computational predictions in the various solvents match both qualitatively and quantitatively, within experimental uncertainty, with the experimental data taken by Vir et al.^4,10^ (yellow crosses and yellow lines). These experimental values have been obtained in bulk through the high-temperature PLP–SEC technique^4,10^ and show an activation energy of 19 ± 4 kcal/mol in the temperature range *T* = 383–413 K. The theoretical rate constants from Cuccato et al.,^14^ which have been calculated through gas-phase HTST and B3LYP/6-31+G(d,p) functional, instead align more closely in terms of activation energy (28.0 kcal/mol) with our results in the gas phase. However, a factor >10^3^ quantitative discrepancy can be observed compared to our computational predictions in vacuum as well as the semibatch evaluated rate parameter from Peck et al.^3^ (red triangle) and a factor >10^4^ compared to the data from Vir et al.^4,10^ together with our predictions in water and BA monomer solvent.

**Figure 7:**
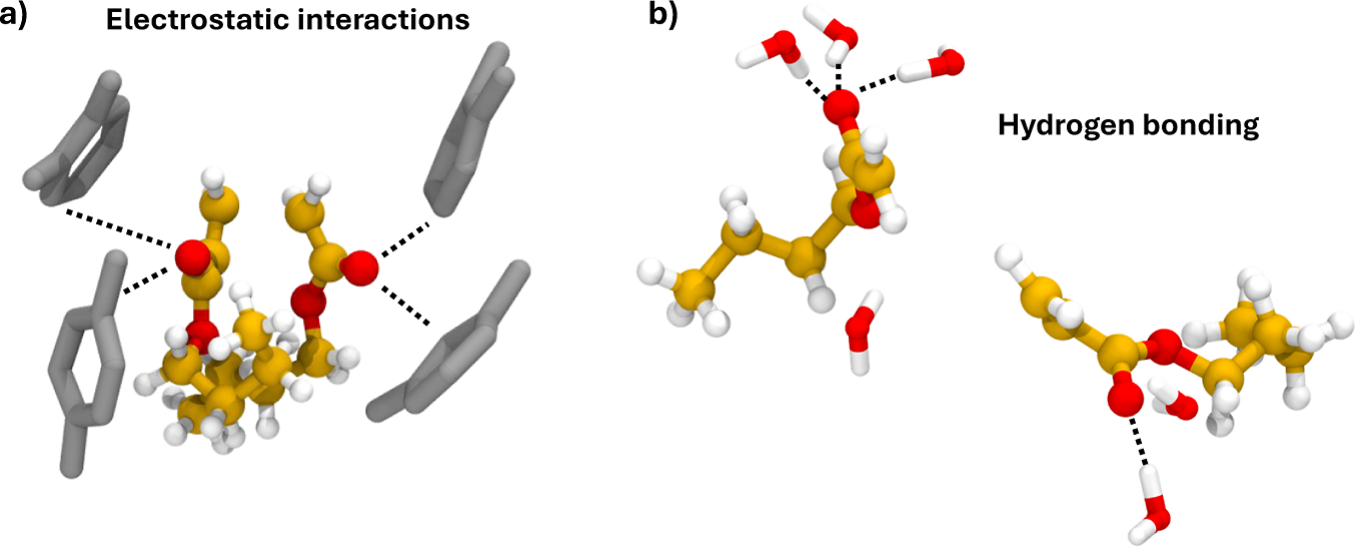
(a) Electrostatic interactions between xylene and BA’s carbonyl groups and (b) hydrogen bonding between the carbonyl groups and water solvent.

The reason for this discrepancy relates to both the error that arises from the relatively small basis set (6-31+G(d,p)) adopted in ref (14) and from neglecting the presence of the solvent. Our results show that, in general, the thermal bath with a solvent significantly affects the reactivity, lowering the free energy barrier of activation by ∼4 kcal/mol. In addition, different solvent types may have different effects on reactivity. The β-scission rate constant evaluated in bulk is a factor of 2 higher with respect to the ones calculated in xylene and water. These solvent effects on reactivity have also been observed experimentally regarding the propagation reaction of acrylate polymers.^36,37^ These studies show that the propagation rate constant, *k*_p_, of acrylate polymers increases by a factor of 2 compared to bulk values when alcohols or water are used as solvents. These studies claim that this difference may be caused by the presence of hydrogen bonding effects between the hydroxyl groups of the solvent and the oxygen atom of the carbonyl groups, which weaken the terminal alkyl double bond, thus favoring the addition to a radical.^36,37^ Conversely, hydrogen bonding may hinder the β-scission with respect to bulk conditions because the weaker π bond of the alkyl group that is being formed reduces the driving force of the chemical reaction. However, this explanation does not apply to xylene solvent, which does not form hydrogen bonds but yields the same kinetic constant as water solvent; therefore, other stabilization effects can be involved in the interactions with aromatic solvents, such as electrostatic interactions between the positively charged hydrogens of the aromatic rings and the negatively charged carbonyl double bond. Such effects can be visualized by analyzing snapshots from our simulations, which are shown in Figure 7.

These results support the hypothesis that an explicit treatment of the solvent is crucial for determining accurate rate parameters in condensed phase, even if the solvent does not directly participate to the chemical reaction investigated.

## 4 Conclusions

This work proposes a theoretical framework for determining the kinetic rate parameters of FRP and pyrolysis processes in solution from accelerated Born–Oppenheimer molecular dynamics simulations. In particular, combining standard metadynamics and MFI allows the achievement of a better compromise between sampling and accuracy of the electronic structure methodology compared to WTMTD. The MFI algorithm opens the possibility to merge multiple independent short simulations into a single refined estimate of the free energy surface associated with the chemical reaction (or a rare event in general), thus significantly reducing the sampling error while maintaining a quantum description of the potential energy. The reactive trajectories so obtained, which account for the specific interactions with different solvent molecules, can be divided into a few snapshots whose potential energy is corrected through highly accurate electronic structure methods, such as ωB97XD/def2-TZVPP, compensating for the errors that may arise from the cheaper potential used in the dynamic sampling and for the lack of quantum effects such as ZPE and tunneling. The correction factor derived in this study from ωB97XD/def2-TZVPP (including the ZPE) of 7.92 kcal/mol, has been applied to all the marginal free energy profiles in the various solvents along the C–C distance, which has been identified as an effective reaction coordinate for the β-scission. The condensed phase rate parameters determined in this work based on the rare events generalized TST^19^ show a factor 10 difference with respect to the results obtained with the same methodology in vacuum. Moreover, the results show a factor 2 difference between the rate parameters in water and xylene (which have the same value) and the one in liquid BA monomer. We have verified through molecular simulations the hypothesis made in previous studies^36−38^ that hydrogen bonding affects the reactivity of acrylate polymers in solution with water. We also hypothesize that aromatic solvents, such as xylene, exert similar stabilization effects as water due to electrostatic interactions between the positively charged hydrogen atoms attached to the aromatic rings and the acrylate’s carbonyl groups. As a result, the β-scission carried out in water and xylene is two times slower with respect to bulk, which better matches with PLP–SEC measurements.^10^ These results support the conclusion that solvents can significantly affect the kinetic rate parameters of radical mechanisms and, therefore, an explicit treatment of the solvent in quantum mechanical simulations is crucial for achieving quantitative estimates of kinetic rate parameters in solution.^39^

## Supporting Information Available

Convergence analysis on the bivariate FES and on the marginal free energy profiles; convergence of the marginal free energy profiles with respect to the integration grid size; and DFT electronic and zero-point energy corrections with different basis sets (PDF)
