# Supplementary material for: Unveiling Solvent Effects on β-Scissions through Metadynamics and Mean Force Integration
Source: J Chem Theory Comput. 2024 Jul 3;20(14):6253–62. doi: 10.1021/acs.jctc.4c00383 (PMC11271823; doi:10.1021/acs.jctc.4c00383)
Supplement: Supplementary file 1 — ct4c00383_si_001.pdf [file ct4c00383_si_001.pdf]

Supplementary Material: Unveiling Solvent Effects on β-Scissions through Metadynamics and Mean Force Integration

Francesco Serse,[1] Antoniu Bjola,[2] Matteo Salvalaglio,[2] and Matteo Pelucchi[1]

[1]*Department of Chemistry Materials and Chemical Engineering, Politecnico di Milano, Piazza Leonardo da Vinci 32, Milan 20133, Italy.*

[2]*Thomas Young Centre and Department of Chemical Engineering, University College London, London, WC1E 7JE, U.K.*

(*francesco.serse@polimi.it)

(*m.salvalaglio@ucl.ac.uk)

FREE ENERGY SURFACE CONVERGENCE AND POTENTIAL ENERGY CORRECTION

All the simulation results of this work together with the python codes used for postptocessing and plotting are available in the GitHub repository: https://github.com/Fserse/Kinetics-from-Metadynamics.
This document gathers the supplementary material regarding the convergence of the Helmholtz free energy estimates calculated through standard metadynamics and Mean Force Integration. Section A shows the results obtained at T=410K in vacuum and in the various solvents analized in this work, namely BA monomer, xylene and water. Section B, reports the marginal free energy pofiles along the effective reaction coordinate (C-C distance) and the related global convergence of the bootstrap error in the same conditions as the two-dimensional case. Finally, Section C reports the convergence of the marginal free energy profile in just one case with respect to the number of gridpoints used for discretizing the integration domain. Finally, the electronic energies at 0 K including the zero-point energy calculated with the unrestricted ωB97XD functional are summarized in Table S1 of Section D. The correction factor employed for the calculation of the rate parameters is the one with the def2-TZVPP basis set, which has been reported to have an error within 2.3 kcal/mol [1].

A. TWO DIMENSIONAL FREE ENERGY

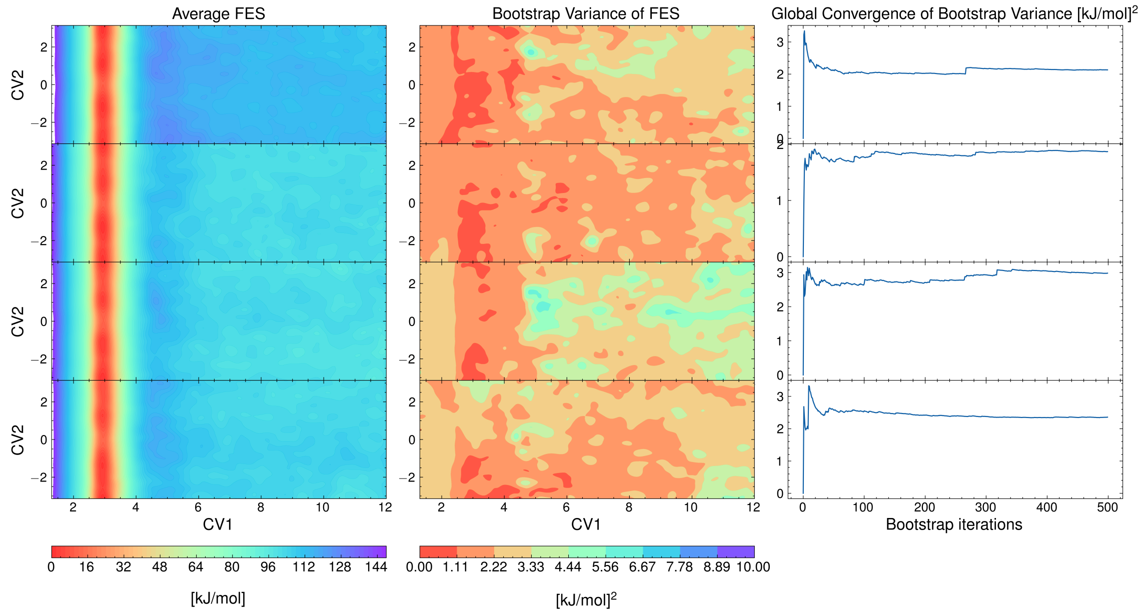

Fig. S1: Twodimensional free energy surfaces, twodimensional bootstrap sample variance, global convergence of the bootstrap standard error for simulations in gas phase, in BA solvent, in xylene solvent and in water at T = 410 K, in order from top to bottom.

B. ONE DIMENSIONAL MARGINAL FREE ENERGY

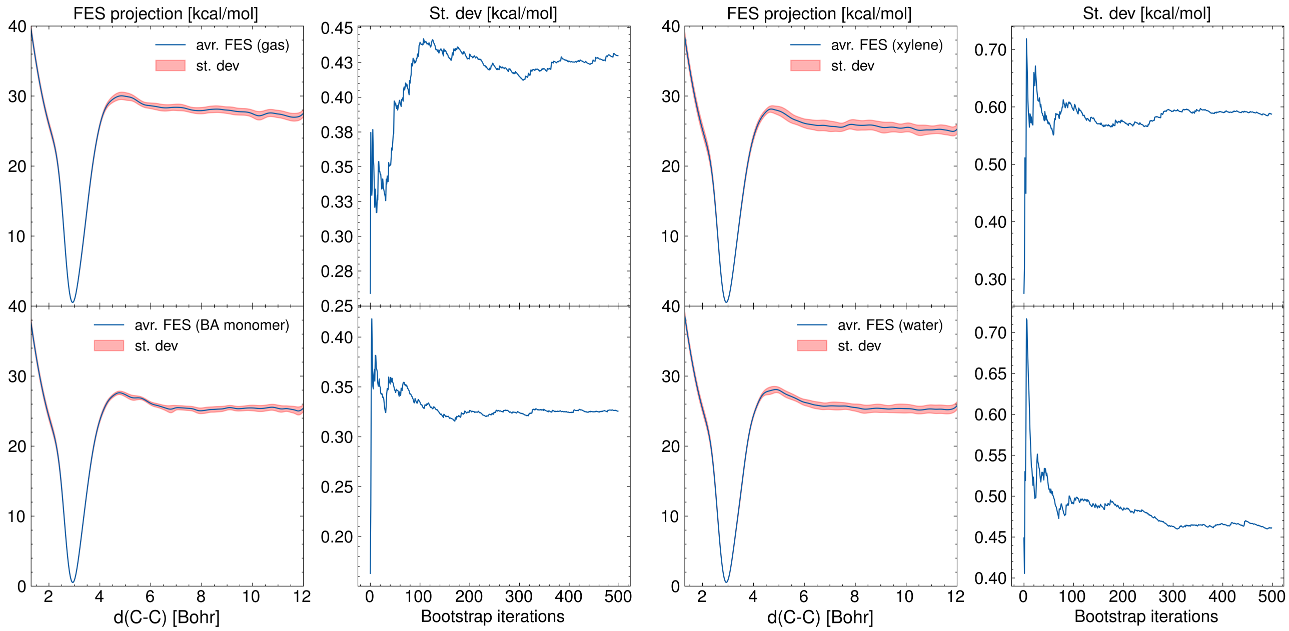

Fig. S2: Convergence analysis of the global bootstrap standard deviation of the marginal free energy profiles along the effective reaction coordinate d_{C-C}.

C. CONVERGENCE WITH RESPECT TO GRID SIZE

The relative error flattens for grids with 800 points between 0 and 12 Bohr.

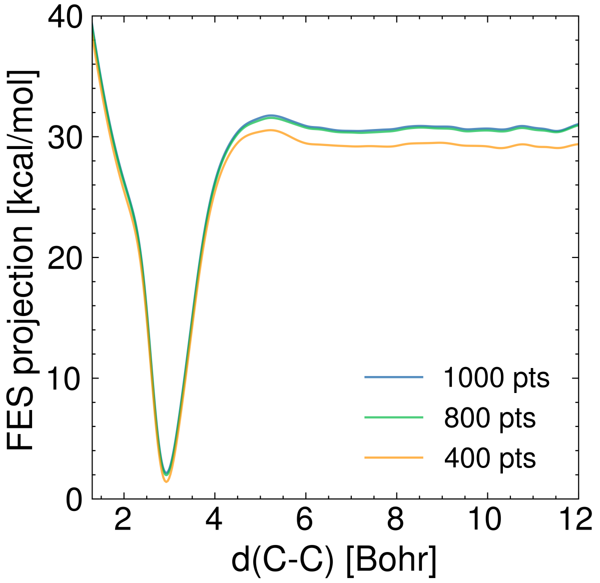

Fig. S3: Convergence analysis of the marginal free energy profile with respect to the number of grid points used for the integration.

D. POTENTIAL ENERGY AND ZERO-POINT ENERGY CORRECTION

TABLE S1: Comparison of absolute potential energy of the reactant dimer radical (R) and the transition state (TS) in vacuum including the zero-point energy with the uωB97XD. The potential energy correction employed for the calculation of rate constants is the one calculated with uωB97XD/def2-TZVPP.

Method	$E_R + ZPE_R$ [Hartree]	$E_{TS} + ZPE_{TS}$ [Hartree]	ΔE [kcal/mol]
uωB97XD/def2-TZVPP	-809.845011	-809.807448	23.57
uωB97XD/6-311+G(d,p)	-809.749844	-809.712612	23.36

[1] Mardirossian, N.; Head-Gordon, M. Thirty years of density functional theory in computational chemistry: an overview and extensive assessment of 200 density functionals. *Molecular Physics* **2017**, *115*, 2315–2372.
